# Long‐term outcomes of ‘temporary’ defunctioning in patients with severe perianal Crohn's disease

**DOI:** 10.1111/codi.17289

**Published:** 2025-01-30

**Authors:** M. A. J. Becker, A. J. M. Pronk, K. Gecse, R. Hompes, W. A. Bemelman, C. J. Buskens

**Affiliations:** ^1^ Tytgat Institute for Liver and Intestinal Research, Amsterdam UMC University of Amsterdam Amsterdam The Netherlands; ^2^ Department of Surgery Amsterdam UMC, Location AMC Amsterdam The Netherlands; ^3^ Department of Gastroenterology Amsterdam UMC, Location AMC Amsterdam The Netherlands

**Keywords:** Crohn's disease, defunctioning ostomy, perianal fistula

## Abstract

**Aim:**

This study aimed to analyse long‐term outcomes in patients undergoing temporary faecal diversion for therapy‐refractory Crohn's perianal fistulas.

**Methods:**

In this retrospective study, Crohn's patients who underwent defunctioning for perianal disease between 2012 and 2022 were included. The primary endpoints were successful ostomy reversal and proctectomy/proctocolectomy rates. Secondary endpoints were clinical fistula closure, development of proximal colonic disease recurrence, and the influence of medication, patient and disease characteristics on ostomy reversal.

**Results:**

In total, 53 patients were included, 35 colostomies and 18 ileostomies. Previous L2 disease was more frequently seen in the ileostomy group (colostomy 29%, ileostomy 83%; *P* = 0.004). Clinical closure of the fistula was seen in 26%. 21% (11/53 patients) underwent an attempt at ostomy reversal during a median overall follow‐up of 90.1 months (interquartile range 17.5–82.5) of which nine ostomies (colostomy 23%, ileostomy 6%; *P* = 0.244) were closed successfully. In 35%, a proctectomy/proctocolectomy was required to control ongoing perianal sepsis. Factors associated with ostomy reversal were fistula closure (*P* < 0.001) and L1/L3 disease (*P* = 0.043). In patients with ostomy reversal attempt (*n* = 11), successful reversal was associated with colostomy (*P* = 0.055) and use of anti‐tumour necrosis factor (anti‐TNF) (*P* = 0.055) despite being previously classified as anti‐TNF refractory.

**Conclusion:**

Ostomy reversal rates are low, so defunctioning ostomies should be carefully considered because in most patients the ostomy will be permanent. In one‐third of the patients, a proctectomy/proctocolectomy is required to treat ongoing perianal sepsis. If a patient is eligible for ostomy reversal, reversal should preferably be done under anti‐TNF to optimize chances of success.

## INTRODUCTION

Up to one‐third of the patients with Crohn's disease (CD) will develop one or more perianal fistulas within 20 years after diagnosis [1, 2]. These perianal fistulas have a negative impact on the quality of life due to pain and discharge and are generally considered as one of the biggest unmet needs in CD [3]. Optimal care for Crohn's perianal fistulas includes a combination of medical therapies (anti‐tumour necrosis factor [TNF]) and surgical therapies (e.g., ligation of the intersphincteric fistula tract, endorectal advancement flap or stem cell injections) [4]. However, in many patients these methods are insufficient, and epidemiological studies suggest that up to 50% will require faecal diversion [5]. In two‐thirds of patients, faecal diversion will reduce symptoms [6]. Generally, patients are counselled for a temporary ostomy. However, the chances of ostomy reversal are not high, with reversal rates ranging from zero to 47% [7]. In addition, according to a systematic review by Singh et al. [8], ostomy reversal was successful in less than half of the patients, with a significant proportion of patients requiring additional proctectomy with a permanent ostomy [8, 9, 10].

When the severity of the perianal disease warrants defunctioning, there are two options, an ileostomy or a sigmoid colostomy. The latter is generally preferred when the proximal colon is not affected as a colostomy produces more solid stool, empties less frequently with less skin irritation, and overall is easier to manage. de Buck van Overstraeten et al. [11] demonstrated that 90% of patients undergoing proctectomy with end colostomy for anorectal CD developed proximal colonic disease recurrence within 1 year. That study therefore suggested that an ileostomy should be considered even when the colon is not affected. In contrast, a study by Lightner et al. [12] showed that colonic recurrence was observed in only a minority of patients after proctectomy (29%). Patients requiring proctectomy for persistent perianal sepsis represent a specific group with progressive disease despite defunctioning, currently considered as a class 2c in the new TOpClass fistula classification system described by Geldof et al. [13].

The aim of this study was to analyse long‐term outcomes of defunctioning ileostomy or colostomy for perianal disease. Patient and disease characteristics were assessed and correlated with (successful) reversal rates or the need for additional resection(s) to improve counselling of our patients with severe perianal disease.

## METHODS

### Study design

In this retrospective cohort study, all patients ≥18 years old with Crohn's perianal fistulas treated with a defunctioning ostomy between January 2012 and May 2022 were included. Patients without CD, ≤18 years old, cryptoglandular fistulas and rectovaginal fistulas were excluded. No ethical approval was needed for this study, confirmed by the Medical Ethical Committee at the Amsterdam UMC. All eligible patients were able to withdraw permission for data collection through an objection form.

### Patient demographics and outcome variables

Patient demographics including sex, age, smoking history, duration of CD, location of CD according to the Montreal classification, use of medication, previous perianal and abdominal surgery, previous ostomy and type of ostomy were collected from medical notes. Outcome variables included fistula closure, ostomy reversal, proximal luminal colonic recurrence, the need for proctectomy or proctocolectomy, permanent defunctioning and additional surgical resections during follow‐up.

### Primary and secondary endpoints

The primary endpoint was successful ostomy reversal, defined as ostomy reversal without the creation of a new ostomy. In addition, the subsequent proctectomy or proctocolectomy rate was determined for both ostomies. Secondary endpoints were fistula closure, colonic recurrence (colonic involvement proximal to the ostomy) after creation of a colostomy, colectomy rates, and the influence of medication, patient and disease characteristics on ostomy reversal. Clinical closure of the fistula was based on physical examination. A fistula was clinically closed when the external opening was closed without discharge of faeces or pus upon pressure.

### Statistical analysis

Descriptive statistics were used to summarize patient characteristics; non‐parametric data were presented as median and interquartile range (IQR) and categorical data were presented as frequencies and percentages. Patient demographics, primary and secondary endpoints were analysed using the Mann–Whitney *U* test, chi‐squared test and Fisher's exact test. Kaplan–Meier analyses was used to calculate the ostomy defunctioning time. A two‐tailed *P* value of <0.05 was considered statistically significant and statistical analyses were performed using IBM SPSS Statistics version 28.0 for Windows.

## RESULTS

### Baseline characteristics

In total, 53 patients were included, 35 patients (66%) with a sigmoid (86%) or descending colon (14%) colostomy and 18 patients (33.3%) with an ileostomy (Table 1). Associated previous L2 disease was more frequently seen in the ileostomy group (colostomy 10/35, 29%; ileostomy 15/18, 83%; *P* = 0.004). In the colostomy group, 80% had an end colostomy; for the ileostomy this was 28%. More than 60% of patients who received an ileostomy had (besides perianal fistula(s)) another indication for an ostomy, of which therapy‐refractory colitis was the most common. The median age of all patients at surgery was 33.0 years (IQR 24.5–39.5), with the majority being women (62%). The median CD duration was 12.0 years (IQR 8.0–19.0), and the most common phenotype was L2 (47%). At the time of surgery, 14 patients (26%) were smoking and 32 patients (60%) were using medication, of whom the majority used anti‐TNF (87.5%). Of note, 10/11 (90.1) of the patients not on medication at the time of surgery did receive anti‐TNF in the past. Eighteen patients (34%) had abdominal surgery in the past, of whom 10 patients (56%) underwent an ileocaecal resection with ileocolic anastomosis. A median follow‐up of 90.1 months (IQR 43.8–184.5) was available for the entire study group. One patient died during follow‐up due to an incurable chronic osteomyelitis and recurrent squamous cell carcinoma.

**TABLE 1:** Baseline characteristics.

	Entire study group *n* = 53	Colostomy *n* = 35	Ileostomy *n* = 18	*P*
Male sex, *n* (%)	20 (38%)	11 (31%)	9 (50%)	0.237
Age, years	33.0 (24.5–39.5)	32.0 (24.0–39.0)	33.5 (25.3–41.3)	0.659
Smoking at time of surgery, *n* (%)	14 (26%)	11 (31%)	3 (17%)	0.416
Duration of Crohn's disease, years (IQR)	12.0 (8.0–190)	13.0 (8.0–16.8)	11.0 (6.5–21.5)	0.571
Location of Crohn's disease, *n* (%)				
L1 Ileum	9 (17%)	8 (23%)	1 (5.6%)	0.143
L2 Colon	25 (47%)	11 (31%)	14 (78%)	
L3 Ileum and colon	17 (32%)	14 (40%)	3 (17%)	
L1–L4	1 (1.8%)	1 (2.9%)	0 (0%)	
Previous colonic activity	25 (47%)	10 (29%)	15 (83%)	0.004
Use of medication, *n* (%)				
Anti‐TNF	15 (28%)	9 (26%)	6 (33%)	0.969
Immunomodulators	4 (7.5%)	3 (8.6%)	1 (5.6%)	
Both	13 (25%)	9 (26%)	4 (22%)	
None	11 (21%)	7 (20%)	4 (22%)	
Missing	10 (19%)	7 (20%)	3 (17%)	
Previous abdominal surgery, *n* (%)	18 (34%)	11 (31%)	7 (39%)	
Ileocaecal resection	10 (19%)	8 (23%)	2 (29%)	0.760
Colectomy	5 (9.4%)	1 (2.9%)	4 (57%)	
Appendectomy	2 (3.8%)	2 (5.7%)	0 (0.0%)	
Section caesarean	1 (1.9%)	0 (0.0%)	1 (14%)	
Type of ostomy, *n* (%)				
End	33 (62%)	29 (83%)	5 (28%)	<0.001
Loop	13 (25%)	5 (14%)	8 (44%)	
Unknown	7 (13%)	2 (5.7%)	5 (28%)	

Abbreviations: IQR, interquartile range; TNF, tumour necrosis factor.

### Ostomy reversal

**FIGURE 1:**
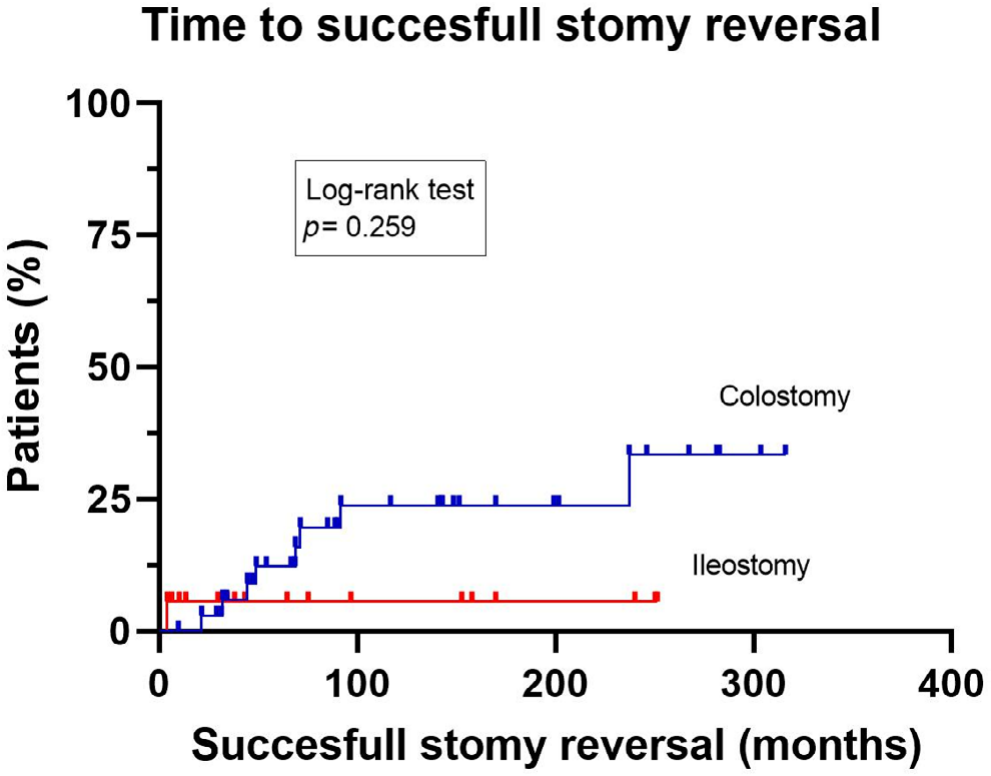
Kaplan–Meier curve with time to successful ostomy reversal (months).

During follow‐up 11 patients (21%) underwent an attempt at ostomy reversal: eight out of 35 (22.9%) patients with a colostomy and three out of 18 (17%) patients with an ileostomy (*P* = 0.933), after a median period of defunctioning of 23.0 months (IQR 12.3–34.5) and 30.0 months (2.0–30.0), respectively (Figure 1). The median follow‐up after ostomy reversal was 52.0 months (IQR 27.0–82.0). Nine ostomies (colostomy 8/36, 22%; ileostomy 1/18, 5.5%; *P* = 0.244) were still successfully reversed after a median follow‐up of 59.0 months (IQR 17.5–82.5).

### Proctectomy and proctocolectomy

In total, 19/53 patients (36%) underwent a proctectomy or proctocolectomy: colostomy 12/19 (63.2%), ileostomy 7/19 (37%), after a median follow‐up of 14.0 months (IQR 9.0–26.0). The most common reason for a proctectomy or proctocolectomy was ongoing fistula disease/perianal sepsis (11/19 patients; 58%) despite defunctioning, followed by therapy‐refractory colitis combined with one or more fistula(s) (5/19 patients; 26%). The other reasons comprised defunctioning proctitis and a carcinoma.

### Fistula closure

In total, 14/53 patients (26%) showed clinical fistula closure after a median defunctioning of 19.0 months (IQR 5.8–48.5) (colostomy 10/35, 29%; ileostomy 4/18, 22%; *P* = 0.748). Four of these 14 patients had clinical closure of the fistula without any surgical intervention; the other 10 patients underwent surgical closure procedure(s). Ten of these 14 patients (71%) had an ostomy reversal attempt, of which eight were successful.

### Proximal colonic recurrence and colectomy

During follow‐up, 16/35 (46%) patients with a colostomy underwent a colonoscopy (for luminal complaints or control) after a median follow‐up of 3.5 months (IQR 1.3–15.0). In 9/16 patients (56%), proximal colonic recurrence was found which was treated medically in all patients (100%) and 2/9 (22.2%) patients needed a colectomy. All patients with proximal disease recurrence were previously diagnosed with L2 disease.

### Factors associated with (successful) ostomy reversal and proctectomy or proctocolectomy

Factors associated with ostomy reversal were fistula closure (ostomy reversal 10/11, 91%; no ostomy reversal 4/42, 10%; *P* < 0.001) and L1/L3 disease (ostomy reversal 9/11, 82%; no ostomy reversal 19/42, 45%; *P* = 0.043) (Table 2). In patients with ostomy reversal attempt (*n* = 11), successful reversal was associated with colostomy (successful reversal 8/9, 89%; no successful reversal 0/2, 0%; *P* = 0.055) and use of anti‐TNF (successful reversal 8/9, 89%; no successful reversal 0/2, 0%; *P* = 0.055); 7/8 successful reversal patients (88%) were previously classified as anti‐TNF refractory (Table 3). A proctectomy or proctocolectomy was associated with persistent active fistulas (proctectomy/proctocolectomy 19/19, 95%; no proctectomy/proctocolectomy 20/34, 59%; *P* < 0.001) and previous L2 disease (proctectomy/proctocolectomy 14/19, 74%; no proctectomy/proctocolectomy 11/34, 32%; *P* = 0.004) (Table 4).

**TABLE 2:** Factors associated with ostomy reversal.

	Ostomy reversal group, *n* = 11	Other patients, *n* = 42	*P*
Male, *n* (%)	6/11 (55%)	14/42 (33%)	0.196
Age, median (IQR)	27.0 (26.0–41.1)	33.0 (23.8–39.3)	0.553
Smoking at time of defunctioning, *n* (%)	5/11 (45%)	9/42 (21%)	0.108
Duration Crohn's disease, median (IQR)	12.0 (8.0–23.0)	12.5 (7.3–18.3)	0.782
L1 or L3 disease, *n* (%)	9/11 (82%)	19/42 (45%)	0.043
Fistula closure, *n* (%)	10/11 (91%)	4/42 (10%)	<0.001
Proctitis, *n* (%)	6/11 (55%)	27/41 (66%)	0.503

Abbreviation: IQR, interquartile range.

**TABLE 3:** Factors associated with successful ostomy reversal.

	Successful ostomy reversal, *n* = 9	Unsuccessful ostomy reversal, *n* = 2	*P*
Male, *n* (%)	5/9 (56%)	1/2 (50%)	1.000
Smoking at time of defunctioning, *n* (%)	5/9 (56%)	0/2 (0%)	0.455
Duration of Crohn's disease, median (IQR)	12.0 (8.0–18.5)	16.5 (8.0)	0.812
Previous L2 disease, *n* (%)	1/9 (11%)	1/2 (50%)	0.345
Use of anti‐TNF at time of reversal, *n* (%)	8/9 (89%)	0/2 (0%)	0.055
Colostomy, *n* (%)	8/9 (89%)	0/2 (0%)	0.011

Abbreviations: IQR, interquartile range; TNF, tumour necrosis factor.

**TABLE 4:** Factors associated with proctectomy or proctocolectomy.

	Proctectomy/proctocolectomy, *n* = 19	Other patients, *n* = 34	*P*
Male, *n* (%)	5/19 (26%)	15/34 (44%)	0.247
Age, median (IQR)	35.0 (25.0–40.0)	31.0 (23.0–38.8)	0.373
Smoking at time of defunctioning, *n* (%)	3/19 (16%)	11/34 (32%)	0.330
Duration of Crohn's disease, median (IQR)	13.0 (10.0–21.0)	9.5 (7.8–15.3)	0.146
Previous L2 disease, *n* (%)	14/19 (74%)	11/34 (32%)	0.005
Persisting active fistulas, *n* (%)	19/19 (100%)	20/34 (59%)	<0.001
Proctitis, *n* (%)	13/19 (68%)	20/34 (59%)	0.382

Abbreviation: IQR, interquartile range.

## DISCUSSION

The present study shows long‐term outcomes (median follow‐up 90 months) after defunctioning in patients with Crohn's perianal fistulas. After a median follow‐up of 23 months, the ostomy was reversed in only 21% of patients. The best predictive factors for ostomy reversal were fistula closure and L1/L3 disease. Overall, ostomy reversal was successful in 17% of all patients, which was associated with a colostomy or the use of anti‐TNF at time of reversal. In 35% of the patients with a defunctioning ostomy, proctectomy or proctocolectomy was required to control ongoing perianal sepsis. Patients with persistent active fistulas and previous L2 disease are more prone to a colectomy or proctocolectomy. During follow‐up only 26% of the patients had clinical closure of the fistula; none of these patients were in the proctectomy/proctocolectomy group. These data provide evidence for clinical decision making and realistic numbers for disease outcome.

As mentioned above, 21% of the patients underwent an ostomy reversal attempt, which was only successful in 17% of the study population. These numbers are in line with previous literature from McCurdy et al. [14] and Marti‐Gallostra et al. [15]. These studies were also conducted in tertiary referral centres and found successful ostomy reversal rates of 21% and 18%, respectively. The study by Régimbeau et al. [16] reported a much higher ostomy reversal rate of 47%. However, the initial fistula healing rate in that study was 65%, much higher compared to the clinical closure rate of 26% in our study, which could explain their higher ostomy reversal rate.

The low ostomy reversal numbers mentioned above show the importance of considering all options before deciding on a defunctioning ostomy. A confounding factor for creating a defunctioning colostomy instead of an ileostomy is the absence of previous colitis [17]. This statement reflects our data, with the majority of patients (56%) receiving an ileostomy instead of a colostomy due to disease activity in the colon. The one patient with successful ostomy reversal received an ileostomy because of perianal sepsis and penetrating disease in the ileocaecal region. During an ileocaecal resection in a second operation, the ileostomy was reversed. This explains the fast continuity repair.

In a recent systematic review by Jew et al. [18], the outcomes of faecal diversion were compared between the pre‐biologic era and the biologic era. The rates of successful restoration were 24% in the pre‐biologic era and 17% in the biologic era, which is consistent with our findings (17%). Similarly, the necessity for completion proctectomy was comparable, with rates of 31% in our study versus 35% in the biologic era and 38% in the pre‐biologic era.

In our case series 94% of the patients received anti‐TNF in the past. However, this was not sufficient for fistula closure and therefore patients were classified as anti‐TNF refractory. In this study, we found that the use of anti‐TNF during ostomy reversal was associated with successful reversal, even when patients were classified anti‐TNF refractory in the past. Our data suggest that, although anti‐TNF will not always achieve fistula closure, it increases the chances of successful ostomy reversal.

In total, 35% of the patients underwent a proctectomy or proctocolectomy because of ongoing perianal sepsis or therapy‐refractory disease. This rate is comparable with the proctectomy rate of 42% from a systematic review including 413 patients in 12 studies [8]. Persisting fistula under an ostomy negatively affects the quality of life. To improve quality of life and prevent redo surgery, these patients would benefit from a direct proctectomy with permanent ostomy. The new TOpClass classification can be used to classify patients in an early stage. As this classification is associated with disease course, especially patients in class 2c (rapidly progressive disease) who are prone to undergo subsequent proctectomy or proctocolectomy could be counselled proactively towards (procto)colectomy or proctectomy during initial diversion surgery [13]. The high numbers of extensive surgeries show us the need to understand the pathophysiology of perianal fistula and the unmet need for new therapies.

The study by de Buck van Overstraeten et al. [11] showed a 90% proximal disease after faecal diversion. After carefully checking the endoscopic evaluation forms of all our patients, we found that, in all patients with proximal disease after defunctioning, activity was already present in the colon during creation of an ostomy. This is also confirmed by the short interval to recurrent disease, suggesting that diverting the faecal stream does not change the phenotype of CD, but that proximal recurrent disease should be seen merely as an exacerbation of the known phenotype. The question could be raised if ostomy outcomes could be optimized by removing a part of the colon during ostomy creation.

A few limitations need to be considered, which are inherent to the retrospective design and missing data. First, our study was performed with a small group of patients, which can yield statistically insignificant results. However, the number of patients receiving a defunctioning ostomy for Crohn's perianal fistulas is low, so the small group of patients reflects daily practice. Second, this study was performed in a tertiary referral hospital which may skew the patient population towards the most severe phenotypes. Third, clinical assessment of fistula closure can be challenging. However, data originate from a tertiary referral centre with a high number of patients with perianal fistulas, ensuring a consistent and uniform approach of reporting clinical fistula outcomes. Finally, only a small number of patients had endoscopic evaluation after creating a defunctioning colostomy. Therefore, data on the proximal recurrence rates are scarce. However, all patients underwent this endoscopy due to complaints. Therefore, it could be hypothesized that patients who did not have complaints were most likely not to have recurrent disease.

In conclusion, this study demonstrates that ostomy reversal rates are low (21%). In 35% of the patients an additional proctectomy or proctocolectomy is required to treat the ongoing perianal sepsis. Patients with previous colonic activity and persistent active fistulas are more prone to a proctectomy/proctocolectomy. Our data suggest that ostomy reversal should preferably be performed under anti‐TNF treatment even when patients are classified as anti‐TNF therapy refractory in the past. In conclusion, a defunctioning ostomy and especially an ileostomy should be carefully considered and seen as a last resort because in most patients the ostomy will be permanent.

## AUTHOR CONTRIBUTIONS

**M. A. J. Becker:** Conceptualization; investigation; writing – original draft; methodology; visualization; writing – review and editing; project administration; formal analysis; data curation. **A. J. M. Pronk:** Conceptualization; investigation; writing – original draft; methodology; visualization; writing – review and editing; formal analysis; project administration; data curation. **K. Gecse:** Writing – review and editing. **R. Hompes:** Writing – review and editing. **W. A. Bemelman:** Writing – review and editing. **C. J. Buskens:** Writing – review and editing; supervision; methodology; conceptualization; writing – original draft.

## FUNDING INFORMATION

This research received no specific grant from any funding agency in the public, commercial or not‐for‐profit sectors.

## CONFLICT OF INTEREST STATEMENT

Marte A.J. Becker: No conflict of interest. Aagje J.M. Pronk: No conflict of interest. Krisztina B. Gecse has received grants from Pfizer Inc., Celltrion and Galapagos; consultancy fees from AbbVie, Arena Pharmaceuticals, Galapagos, Gilead, Immunic Therapeutics, Janssen Pharmaceuticals, Novartis, Pfizer Inc., Samsung Bioepis and Takeda; and speaker's honoraria from Abbvie, Celltrion, Ferring, Janssen Pharmaceuticals, Novartis, Pfizer Inc., Samsung Bioepis, Takeda and Tillotts. R. Hompes: No conflict of interest. W.A. Bemelman: Received an unrestricted grant from VIFOR, and speaker fees from Galapagos and Olympus. Christianne J. Buskens: Received an unrestricted grant from Boehringer Ingelheim and Roche, and honoraria or speaker fees from Abbvie, Tillotts, Takeda and Janssen.

## ETHICS STATEMENT

This study was conducted as a retrospective analysis using anonymized patient data. According to Dutch law (Medical Research Involving Human Subjects Act, WMO), retrospective studies that do not involve interventions or additional risks for patients are exempt from the requirement for ethical review by a medical ethics committee. Informed consent was not required as no identifiable patient data were used. A notification letter was sent to all patients whose data were included in this study, offering them the opportunity to object to the use of their data. Only data from patients who did not object were included in the analysis.

## Data Availability

The data that support the findings of this study are available on request from the corresponding author. The data are not publicly available due to privacy or ethical restrictions.
